# Admission of patients with infectious disease at the medical admissions ward, Sygehus Thy-Mors – quality-goals

**DOI:** 10.1186/1757-7241-20-S2-P4

**Published:** 2012-04-16

**Authors:** Rita Vosyliene

**Affiliations:** 1 Sygehus Thy-Mors Hospital, Denmark

## Background

In the Electronic Patient Charts at Sygehus Thy-Mors (STM) there is no recording of patient arrival-time, admission-time or time of treatment-start. This makes establishing quality-goals difficult.

## Methods

This study analyses admissions with infectious diseases in the period Jan-Feb 2010 to the medical admissions ward at STM. Especially with respect to establishing quality-goals, for the admission of these patients.

## Results

The study included 93 patients admitted with infections regardless of the suspected diagnosis at the time of referral. It was found that there was a large discrepancy between suspected diagnosis and diagnosis found at the hospital. 58% was admitted with a suspected infection. 63% of the patients over 71 years where admitted with a suspected diagnosis other than infection, i.e. dehydration, apoplexy etc. There where no records of suspicion of SIRS or evaluation of sepsis-criteria even though 54% of the patients matched the criteria. The conclusion is that sepsis and SIRS are grossly underestimated during admission of especially elderly patients with vague symptoms. ABG was ordered in 42% of cases, lactate in 6%. ABG is most frequently ordered in suspected pneumonia or COPD.

## Conclusion

The study establishes three quality-goals: 1) Immediate assessment of SIRS-criteria upon pt’s arrival – especially in elder pt’s with no straightforward symptoms and/or diagnosis; 2) 95% of pt’s meeting the sepsis-criteria upon admission is ordered both ABG and lactate; 3) 85% of pt’s meeting the sepsis-criteria is given i.v. fluids within 30 min. and antibiotics within 60 min.

